# CA19–9 decrease and survival according to platelet level in patients with advanced pancreatic cancer

**DOI:** 10.1186/s12885-019-6078-2

**Published:** 2019-08-30

**Authors:** Y. Chen, Y. R. Wang, G. C. Deng, G. H. Dai

**Affiliations:** 1 Department of Medical Oncology, Chinese People’s Liberation Army (PLA) General Hospital and Chinese PLA Medical School, Beijing, 100853 China; 2 Key laboratory of Carcinogenesis and Translational Research (Ministry of Education/Beijing), Department of Gastrointestinal Oncology, Peking University Cancer Hospital and Institute, 52 Fucheng Road, Haidian District, Beijing, 100142 China

**Keywords:** Pancreatic cancer, CA19–9, Platelet, Prognosis, Chemotherapy

## Abstract

**Background:**

CA19–9 decrease during treatment has been associated with superior survival of pancreatic cancer in several studies. The evidence to show the correlation of high platelet level with inferior survival is insufficient in pancreatic cancer. It also remains unclear whether the association between CA19–9 decrease and survival was corresponded to different levels of platelet in metastatic pancreatic cancer.

**Methods:**

We measured CA19–9 serum concentration and platelet level at baseline and after the second cycle of chemotherapy for 200 advanced pancreatic cancer patients. A Cox proportional hazards model was used to compute mortality hazard ratios (HRs) for CA19–9 decrease, adjusting for potential confounders, including age, sex, KPS, prediagnosis body mass index, Diabetes Mellitus, tumor location, first-line chemotherapy regimen, and radiotherapy.

**Results:**

We found that the association of CA19–9 decrease with superior overall survival was stronger in advanced pancreatic cancer with a low level of platelet (*P*_interaction_ <  0.001) compared with intermediate and high level of platelet. Multivariable-adjusted hazard ratios per unit decrease of CA19–9 change was 0.45 [95% confidence interval (CI), 0.33 to 0.62] in cases with low platelet level, 0.74 (95% CI, 0.50 to 1.09) in cases with intermediate platelet level, and 0.94 (95% CI, 0.74 to 1.10) in cases with high platelet level. A similar differential association was found between CA19–9 decrease and progression-free survival in strata of platelet level (*P*_interaction_ = 0.034).

**Conclusion:**

The association of CA19–9 decrease with superior pancreatic cancer survival appeared to be pronounced in patients with a low platelet level. This finding could provide supports for the underlying mechanisms of CA19–9 involved in platelet / tumor cell interaction.

**Electronic supplementary material:**

The online version of this article (10.1186/s12885-019-6078-2) contains supplementary material, which is available to authorized users.

## Background

Pancreatic cancer is the fourth leading cause of cancer deaths in United States for both men and women and will be the second leading cause by 2030 [1]. Carbohydrate antigen 19–9 (CA19–9) is a sialylated blood group antigen, first defined by Koprowski et al in 1979 [2]. To date, CA19–9 is a widely studied biomarker for diagnosis and prognosis prediction of pancreatic cancer [3, 4]. In general, lower versus higher CA19–9 levels at baseline and decreasing versus increasing CA19–9 during therapy are linked to a superior survival [5]. However, Bauer et al reported that a decline of CA19–9 after the second cycle of chemotherapy is not predictive of improved mOS or mTTP in advanced pancreatic cancer patients who receive gemcitabine-containing chemotherapy in clinical trials [6].

The contribution of platelets to tumor metastatic has been revealed since 1960s by in vivo experiments [7]. Platelets and their releasates can sustain proliferative signals, resist cell death, induce angiogenesis, activate invasion and metastasis and evade immune detection, support cancer stem cells, and even protect circulating tumor cells [8]. But the association of high platelet level with inferior survival in pancreatic cancer is inconclusive [9, 10]. Miyamoto et al and Miura et al combined platelet, C-reactive protein or CA19–9 to build a new parameter or a scoring system predicting survival [11, 12]. However, it remains uncertain whether the prognostic association of CA19–9 decrease differs by platelets level. We hypothesized that the prognostic association of CA19–9 decrease might be stronger in pancreatic cancer patients with a low platelet level than in pancreatic cancer patients with a relatively high platelet level.

To test this hypothesis, we used data on CA19–9 decreased level, pancreatic cancer characteristics, and patients’ clinical outcomes in Chinese People’s Liberation Army (PLA) General Hospital and examined the prognostic association of CA19–9 decrease in strata of platelet level.

## Methods

### Study population

This was a retrospective study approved by the ethics committee of Chinese People’s Liberation Army (PLA) General Hospital. Patients diagnosed with advanced pancreatic cancer and admitted for chemotherapy were included for analysis from August 1, 2010 to December 1, 2016. Follow-up evaluations were performed every 6 months. Dates of death were obtained from the document or telephone calls follow-up. Study physicians reviewed medical records and recorded clinicopathological features. The inclusion criteria were: (1) patients were cytological or histologically confirmed advanced pancreatic cancer; (2) patients received at least 2 cycles (6 weeks) of first-line chemotherapy; (3) normal bone marrow function; (4) normal hepatic and renal function; (5) patients with a Karnofsky performance status (KPS) score of 70 or more; (6) no history of previous chemotherapy for malignant disease. Exclusion criteria: (1) incomplete data of baseline CA19–9 or CA19–9 after the second cycle of chemotherapy or baseline platelet count; (2) lost follow-up. Total 200 patients who had a baseline and a week-6 measurement of CA19–9 were eligible for analysis. Patients were observed until death or June 5, 2018, whichever came first.

### Assessment of CA19–9 and other hematological examination

Laboratory data, including CA19–9, platelet count were obtained within 1 week before patients receive chemotherapy and every 3 weeks thereafter. The upper limit of normal CA19–9 was 37 U/ml and the maximum was 20,000 U/ml. The decreased CA19–9 was defined as the concentration measured at week-6 minus the baseline value and then divided by the baseline value {([CA19–9 at week-6]-[CA19–9 at baseline]) / (CA19–9 at baseline)}. Initially, we included all advanced pancreatic cancer patients in our primary analysis. Then, we included patients with a CA19–9 level greater than 37 U/ml as a sensitivity analysis. We primarily used platelet level as a continuous variable (scale 100–558 × 10^9^/L) in survival analyses. To display our results, we categorized platelet level into three groups, namely platelet level-low (< 166 × 10^9^/L), intermediate (167–220 × 10^9^/L), and high (≥ 221 × 10^9^/L). Overall survival (OS) was defined as the time from date of initial treatment to death. Progression-free survival (PFS) was defined as the time from date of initial treatment to disease progress or death. Censoring occurred if patients were still alive at last follow up.

### Statistical analysis

Outcome end points were OS and PFS. Our primary hypothesis testing was an assessment of a statistical interaction (using the Wald test for the cross-product) between CA19–9 decreases level (continuous) and baseline platelet level (continuous) in the multivariable-adjusted Cox proportional hazards regression model. We initially included the variables of age at diagnosis (continuous), sex, KPS (70–80 vs. 90–100), prediagnosis body mass index (continuous), location (head vs. body/tail), first-line chemotherapy regimen (Gemcitabine monotherapy vs. Gemcitabine plus 5-Fu vs. Gemcitabine plus nab-PTX vs. Gemcitabine plus DDP vs. Nab-PTX plus S-1), and radiotherapy (yes vs. no) and conducted backward elimination with a threshold *P* of 0.05 to select variables for the final model. Disease stage (locally advanced vs. metastatic) was used as a stratifying variable using the “strata” option in SPSS in the Cox model. For cases with missing information in any of the categorical covariates [KPS (2.6%), diabetes mellitus (3.1%), disease stage (1.5%), chemotherapy regimen (2.3%), radiotherapy (2.9%)], we included these cases in the majority category of a given covariate. Cumulative survival probabilities were estimated using Kaplan-Meier method and compared using log-rank test. All statistical analyses were performed using SPSS (Version 20) and software packages R (http://www.r-project.org, The R Foundation) and EmpowerStats (http://www.empowerstats. com, X&Y Solutions, Inc., Boston, MA). All *P* values were two-sided.

## Results

We retrospectively included 200 advanced pancreatic cancer patients with available CA19–9 levels at baseline and week-6. During the median follow-up time of 9.8 months for all censored patients, there were 155 deaths. The median overall survival (OS) in this group of patients was 7.91 months (95% CI, 2.47–21.19) and median progression-free survival (PFS) was 5.29 months (95%CI, 1.48–17.28). Of the 200 patients, the median baseline CA19–9 level was 1364.5 U/ml, median change after 6 weeks was decreased by 22% compared with baseline level (Table 1).

**Table 1:** Characteristics of advanced pancreatic cancer patients with baseline and week-6 measurements

Characteristic^*^	*N* = 200
Age	
Median, IQR	55 (50–62)
Sex (Male/Female)	
Male	127 (63.5%)
Female	73 (36.5%)
KPS	
90–100	169 (84.5%)
70–80	31 (15.5%)
Prediagnosis body mass index	
Median, IQR	22.8 (21.0–25.0)
Diabetes Mellitus	
Absent	152 (76.0%)
Present	48 (24.0%)
Location	
Head/Uncinate	73 (36.5%)
Body/tail	124 (62.0%)
Overlapping sites	3 (1.5%)
Stage	
III	22 (11.0%)
IV	178 (89.0%)
Liver metastasis	
Absent	53 (26.5%)
Present	147 (73.5%)
Baseline CA19–9, U/ml	
Median, IQR	1364.5 (203.6–9094.3)
CA19–9 change†	
Median, IQR	−0.22 ([− 0.60]-0.088)
Baseline Platelet (×10^9^/L)	
Median, IQR	195 (153.0–235.3)
Chemotherapy regimen	
Gemcitabine monotherapy	34 (17.0%)
Gemcitabine plus 5-Fu	18 (9.0%)
Gemcitabine plus nab-PTX	16 (8.0%)
Gemcitabine plus DDP	9 (4.5%)
Nab-PTX plus S-1‡	123 (61.5%)
Radiotherapy	
Yes	16 (8.0%)
No	184 (92.0%)

* Percentage indicates the proportion of patients with a specific clinical, pathologic, or molecular characteristic among all patients

† CA19–9 change = ([CA19–9 at week-6]-[CA19–9 at baseline]) / (CA19–9 at baseline)

Abbreviations: KPS, Karnofsky Performance Status; IQR, Inter Quartile Range

‡ This chemothrapy regimen is a phase II clincial trial conducted in our institute (NCT02124317)

By median value 1365 U/ml for baseline CA19–9, both PFS and OS were inferior for patients with a higher baseline CA19–9 compared with lower CA19–9 level. We observed the same trend when we used the cut-off of 500 or 1000 U/ml for baseline CA19–9 in survival analysis (Additional file 1: Table S1). Patients with per unit decrease (100% decrease after week 6 compared with baseline) in CA19–9 had a significant longer overall survival (multivariable-adjusted HR, 0.84; 95%CI: 0.75–0.94) (Table 2). However, the association between baseline platelet level and patients survival seems null in our data set, which is consistent with previous studies [9, 10].

**Table 2:** Survival by category of CA19–9 and platelet level in advanced pancreatic cancer patients

	No. of cases	No. of events	PFS			OS	
			Univariate HR (95% CI)	Multivariate HR^*^ (95% CI)	No. of events	Univariate HR (95% CI)	Multivariate HR^*^ (95% CI)
Baseline CA19–9 level							
< 1365 U/ml (median)	100	90	1 (reference)	1 (reference)	74	1 (reference)	1 (reference)
≥ 1365 U/ml	100	93	1.53 (1.14–2.06)	1.76 (1.27–2.43)	81	1.61 (1.17–2.21)	1.93 (1.34–2.77)
Change in CA19–9 level at week-6†							
Per unit decreases of CA19–9	200	183	0.86 (0.78–0.95)	0.86 (0.78–0.95)	155	0.87 (0.79–0.96)	0.84 (0.75–0.94)
Baseline Platelet level							
Tertile 1 (lowest)	66	59	1 (reference)	1 (reference)	50	1 (reference)	1 (reference)
Tertile 2	67	64	1.28 (0.90–1.83)	1.21 (0.83–1.77)	52	1.15 (0.78–1.70)	1.02 (0.67–1.54)
Tertile 3 (highest)	67	60	0.93 (0.65–1.34)	0.95 (0.64–1.41)	53	0.88 (0.60–1.31)	0.86 (0.56–1.31)

* The multivariable, stage (stage III vs. stage IV)-stratified Cox regression model initially included age (continuous), sex (female vs. male), KPS (70–80 vs. 90–100), prediagnosis body mass index (continuous), tumor location (head/uncinate vs. body/tail vs. overlapping sites), diabetes mellitus (absent vs. present), chemotherapy regimen (Gemcitabine monotherapy vs. Gemcitabine plus 5-Fu vs. Gemcitabine plus nab-PTX vs. Gemcitabine plus DDP vs. Nab-PTX plus S-1), and radiotherapy (yes vs. no). A backward elimination with a threshold of *P* = 0.05 was used to select variables in the final models

† CA19–9 change = ([CA19–9 at week-6]-[CA19–9 at baseline]) / (CA19–9 at baseline); per unit equals a 100% decrease

Abbreviations: CI, confidence interval; HR, hazard ratio; KPS, Karnofsky Performance Status; PFS, progression-free survival; OS, overall survival

We observed a statistically significant interaction between CA19–9 decrease and platelet level in overall survival analysis (*P*_interaction_ <  0.001; Table 3). CA19–9 decrease was associated with longer OS in patients with low platelet level (multivariable adjusted HR, 0.45; 95%CI: 0.33–0.62), but not in patients with intermediate (multivariable adjusted HR, 0.74; 95%CI: 0.50–1.09) or high platelet level (multivariable adjusted HR, 0.94; 95%CI: 0.74–1.10). The results were similar for PFS (*P*_interaction_ = 0.034). In addition, we did a sensitivity analysis by excluding patients with a CA19–9 < 37 U/ ml (*n* = 14). The interaction between the prognostic association of CA19–9 decrease and baseline platelet level was even stronger (*P*_interaction_ = 0.012, PFS; *P*_interaction_ <  0.001, OS; Additional file 1: Table S2 and Table S3). Furthermore, we examined the prognostic interaction of CA19–9 decrease with baseline neutrophils, lymphocytes, neutrophils and lymphocytes ratio (NLR), and platelets and lymphocytes ratio (PLR) in relation to overall survival, the results were null (Additional file 1: Table S4).

**Table 3:** Changes in the CA19–9 and survival in relation to baseline platelet level in advanced pancreatic cancer patients

	No. of cases	No. of events	PFS per unit decrease of CA19–9†		No. of events	OS per unit decrease of CA19–9†	
			Univariate HR (95% CI)	Multivariate HR^*^ (95% CI)		Univariate HR (95% CI)	Multivariate HR^*^ (95% CI)
Total patients	200	183	0.86 (0.78–0.95)	0.85 (0.77–0.94)	155	0.87 (0.79–0.96)	0.83 (0.74–0.93)
Baseline platelet level							
Tertile 1 (lowest)	66	59	0.69 (0.55–0.85)	0.51 (0.38–0.69)	50	0.57 (0.45–0.73)	0.45 (0.33–0.62)
Tertile 2	67	64	0.75 (0.50–1.14)	0.85 (0.54–1.35)	52	0.72 (0.51–1.03)	0.74 (0.50–1.09)
Tertile 3 (highest)	67	60	0.90 (0.79–1.03)	0.88 (0.75–1.02)	53	0.93 (0.80–1.08)	0.94 (0.74–1.10)
*P*_interaction_‡			0.026	0.034		0.001	< 0.001

* The multivariable, stage (stage III vs. stage IV)-stratified Cox regression model initially included age (continuous), sex (female vs. male), KPS (70–80 vs. 90–100), prediagnosis body mass index (continuous), tumor location (head/uncinate vs. body/tail vs. overlapping sites), diabetes mellitus (absent vs. present), chemotherapy regimen (Gemcitabine monotherapy vs. Gemcitabine plus 5-Fu vs. Gemcitabine plus nab-PTX vs. Gemcitabine plus DDP vs. Nab-PTX plus S-1), and radiotherapy (yes vs. no). For total patients, we additionaly adjusted for platelet level (ordinal: tertile 1, 2, 3). A backward elimination with a threshold of *P* = 0.05 was used to select variables in the final models

† CA19–9 change = ([CA19–9 at week-6]-[CA19–9 at baseline]) / (CA19–9 at baseline); per unit equals a 100% decrease

‡ *P*_interaction_ was calculated using the Wald test for the cross-product of prediagnosis platelet level (continuous) and CA19–9 decrease (continuous) in Cox regression model

Abbreviations: CI, confidence interval; HR, hazard ratio; KPS, Karnofsky Performance Status

**Fig. 1:**
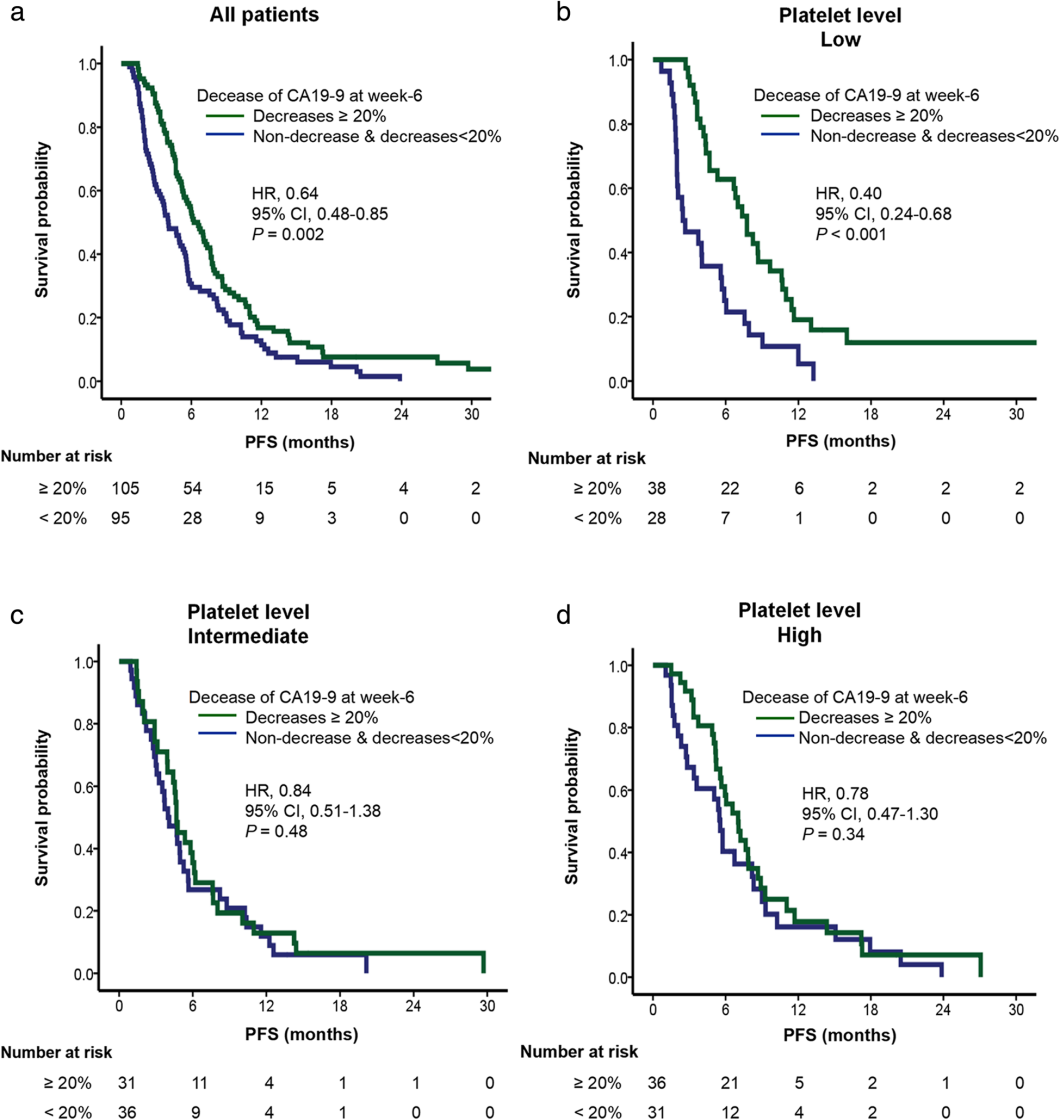
Kaplan-Meier curves of PFS according to CA19–9 decrease in strata of baseline platelet level (**a**. All patients, **b**. Patients with low platelet level, **c**. Patients with intermediate platelet level, **d**. Patients with high platelet level). The HR were calculated using univariate Cox regression model compared patients who experienced a CA19-9 decrease at week-6 above 20% with patients who did not

**Fig. 2:**
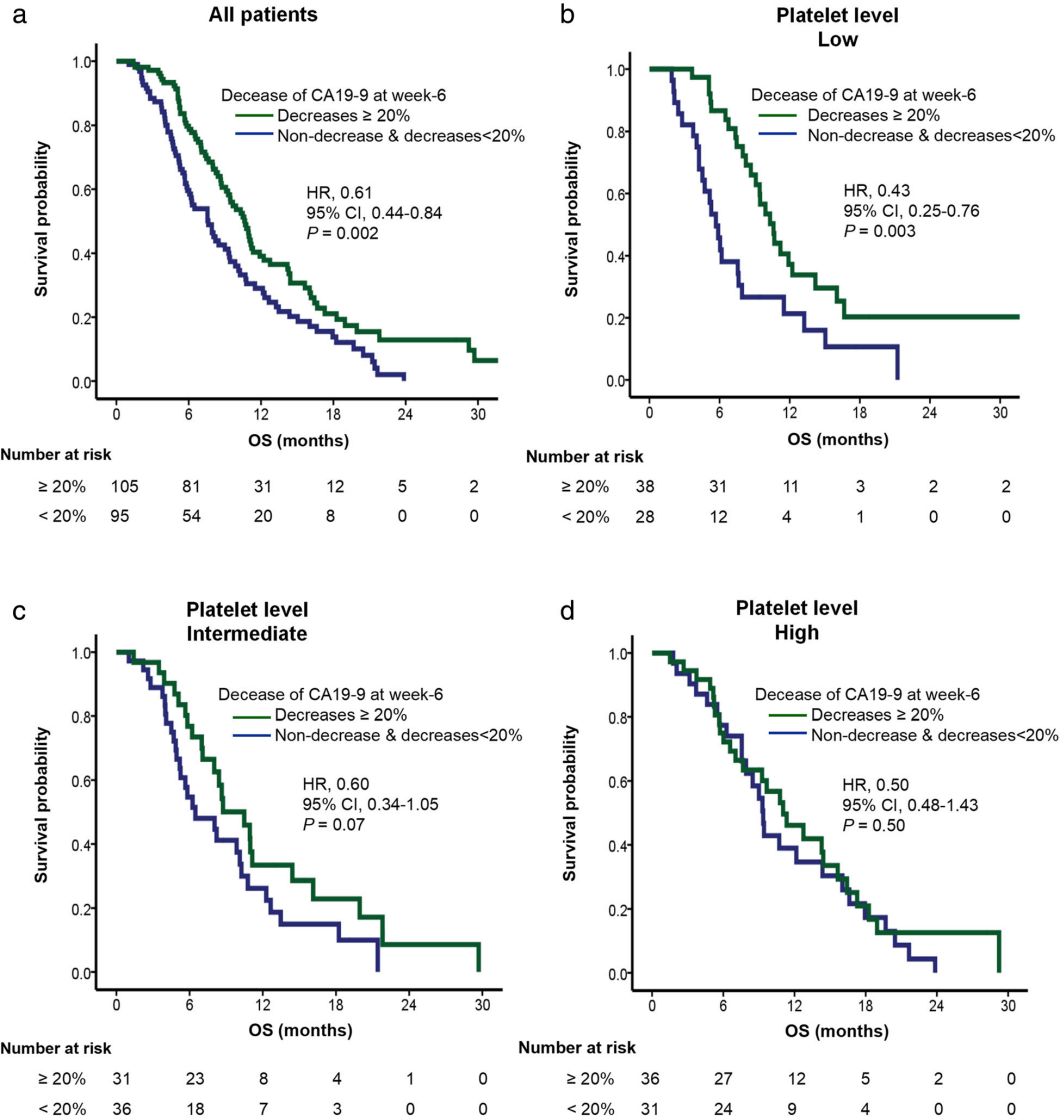
Kaplan-Meier curves of OS according to CA19–9 decrease in strata of baseline platelet level (**a**. All patients, **b**. Patients with low platelet level, **c**. Patients with intermediate platelet level, **d**. Patients with high platelet level). The HR were calculated using univariate Cox regression model compared patients who experienced a CA19-9 decrease at week-6 above 20% with patients who did not

In Kaplan-Meier survival analysis, CA19–9 decrease by 20% was associated with longer OS and PFS (*P* <  0.005) in patients with low platelet level, but not in patients with intermediate or high platelet level (*P* > 0.05; Figs. 1 and 2). Patients with ≥20% decline in CA19–9 after 2 cycles of chemotherapy had significantly better outcomes than those who did not (median OS and PFS of 10.61 and 7.75 months vs 5.68 and 2.46 months; *P* < 0.005) in low platelet level, but not in intermediate or high platelet level (Table 4).

**Table 4:** Analysis of PFS and OS based on CA19–9 change at week-6 in relation to baseline platelet level in advanced pancreatic cancer patients

		No. of cases	PFS		OS	
			Median (95% CI)	*P* value*	Median (95% CI)	*P* value*
All patients	Decreases ≥20%	105	4.04 (2.80–5.28)	0.002	7.62 (5.83–9.41)	0.002
	Non-decrease & decreases< 20%	95	6.57 (5.43–7.71)		10.68 (9.33–12.03)	
Baseline platelet level						
Tertile 1 (lowest)	Decreases ≥20%	38	2.46 (0.29–4.64)	< 0.001	5.68 (4.60–6.77)	0.003
(100–166) × 10^9^/L	Non-decrease & decreases< 20%	28	7.75 (6.11–9.40)		10.61 (8.97–12.26)	
Tertile 2	Decreases ≥20%	31	3.98 (2.48–5.47)	0.48	6.47 (3.39–9.56)	0.07
(167–220) × 10^9^/L	Non-decrease & decreases< 20%	36	4.67 (3.77–5.56)		10.48 (7.59–13.37)	
Tertile 3 (highest)	Decreases ≥20%	36	5.55 (4.82–6.29)	0.34	9.36 (8.21–10.51)	0.50
(221–558) × 10^9^/L	Non-decrease & decreases< 20%	31	7.03 (5.37–8.69)		11.04 (7.45–14.63)	

* P value was calculated by log-rank test

## Discussion

In this study, we found the association of CA19–9 decrease with pancreatic cancer superior survival was stronger for patients of low platelet level, compared with patients of intermediate or high platelet level. To our knowledge, this is the first study to evaluate the prognostic value of CA19–9 decrease in strata of platelet level. Although validation in independent datasets is needed, our findings provide the first line of population-based evidence for the role of platelet in mediating the influence of CA19–9 decrease in the progression of pancreatic carcinomas. Although we should interpret the results cautiously, platelet level can potentially be used as an additional biomarker combined with CA19–9 decrease during treatment in prognosis prediction.

Chiorean et al reported that any CA19–9 decrease at week-8 could be an early marker for chemotherapy efficacy in patients with metastatic pancreatic cancer [13]. Whereas, Hess et al found that an early decrease in CA 19–9 concentration of at least 50% after two cycles of chemotherapy was not associated with a longer overall survival [14]. The prognostic value of an early CA19–9 decrease in pancreatic cancer is controversial. Therefore, there is a substantial need to better understand if there is any factor could potentially modify the prognostic value of CA19–9 decrease. Recently, platelet count, CA19–9 and other parameters are utilized as new criteria for disease diagnosis, treatment and prognosis prediction [11, 12]. Chemotherapy-induced neutropenia (CIN) is a surrogate prognostic marker validated in various tumors [15, 16]. Our group observed the association of CIN, NLR, and PLR with prognosis in pancreatic cancer [17, 18]. However, no study has evaluated the prognostic effects of CA19–9 in strata of platelet. Our findings supporting the differential prognostic effects of CA19–9 decrease according to different platelet level.

It is interesting to speculate potential mechanisms of interaction between CA19–9 and platelet level. Experimental evidence supports that CA19–9 monosialoganglioside may be involved in platelet/tumor cell interactions, playing an important role in the metastases of colorectal cancer [19]. The experimental results implied that CA19–9 and platelet may have a special interaction, thus platelet level may affect the function or level of CA19–9 in pancreatic cancer. In addition, Woei AJFJ et al proved that the binding of CA19–9 to apomucins was correlated with microparticle-associated Tissue factor (TF) activity [20]. TF expressed by tumor cells triggers the formation of thrombin, which leads to both coagulation and platelet activation [21]. As we all known, platelets support tumor metastasis [22]. Thus, a high platelet level may reverse the prognostic value of CA19–9 decrease on survival. Our data provide clinical evidence for possible synergism of CA19–9 and platelet to tumour progression. This study suggests that CA19–9 decrease may be a stronger prognostic factor in pancreatic cancer patients with a low platelet level.

While we recognize the inherent bias in excluding patients with only a solitary CA19–9 measurement at baseline (follow-up measurements were generally not obtained on these patients for rapid disease progression and/or clinical deterioration), therefore, we included them as non-decrease and the results did not change (data not shown). There are several limitations in our study: First, it was a retrospective study, conducted in a single center. Second, platelet which is a continuous variable categorized for analysis that could generate potential bias. Despite the above limitation in our analysis, the highly statistically significant findings indicate a strong interaction between CA19–9 decrease and baseline platelet level in relation to survival.

In conclusion, we demonstrated a stronger association of CA19–9 decrease with pancreatic cancer overall survival in tumors with low platelet level than in tumors with intermediate / high platelet level. Our data suggest that higher platelet level may attenuate survival benefits associated with CA19–9 decrease. This may serve as a prognostic factor to make key clinical decisions. Given growing popularity of finding prognostic factors in pancreatic cancer, our findings, if validated, may have considerable clinical implications for metastatic pancreatic cancer in the era of chemotherapy.

## Additional file

Additional file 1: **Table S1.** Survival by category of CA19–9 in advanced pancreatic cancer patients. **Table S2.** Survival by category of CA19–9 and platelet level in pancreatic cancer patients (CA19–9 > 37 U/ml). **Table S3**. Changes in the CA19–9 and survival in relation to baseline platelet level in advanced pancreatic cancer patients (CA19–9 > 37 U/ml). **Table S4.** The interaction between changes in CA19-9 and baseline hematimetric variables in survival anlysis. (DOCX 29 kb)

## Data Availability

All the data and materials supporting the conclusions were included in the main paper.

## Abbreviations

CA19–9: Carbohydrate antigen 19–9
CI: Confidence interval
HR: Hazard ratio
KPS: Karnofsky performance status
OS: Overall survival
PFS: Progression-free survival
