# Supplementary material for: CA19–9 decrease and survival according to platelet level in patients with advanced pancreatic cancer
Source: BMC Cancer. 2019 Aug 30;19:860. doi: 10.1186/s12885-019-6078-2 (PMC6716806; doi:10.1186/s12885-019-6078-2)
Supplement: Supplementary file 1 — Table S1. Survival by category of CA19–9 in advanced pancreatic cancer patients. Table S2. Survival by category of CA19–9 and platelet level in pancreatic cancer patients (CA19–9 > 37 U/ml). Table S3. Changes in the CA19–9 and survival in relation to baseline platelet level in advanced pancreatic cancer patients (CA19–9 > 37 U/ml). Table S4. The interaction between changes in CA19-9 and baseline hematimetric variables in survival anlysis. (DOCX 29 kb) [file 12885_2019_6078_MOESM1_ESM.docx]

Table S1. Survival by category of CA19-9 in advanced pancreatic cancer patients

|  |  |  | PFS | |  |  | OS | |
| --- | --- | --- | --- | --- | --- | --- | --- | --- |
|  | No. of cases | No. of events | Univariate  HR (95% CI) | Multivariate  HR^*^ (95% CI) |  | No. of events | Univariate  HR (95% CI) | Multivariate  HR^*^ (95% CI) |
| Baseline CA19-9 level |  |  |  |  |  |  |  |  |
| <1365 U/ml (median) | 100 | 90 | 1 (reference) | 1 (reference) |  | 74 | 1 (reference) | 1 (reference) |
| ≥1365 U/ml | 100 | 93 | 1.53 (1.14-2.06) | 1.76 (1.27-2.43) |  | 81 | 1.61 (1.17-2.21) | 1.93 (1.34-2.77) |
|  |  |  |  |  |  |  |  |  |
| <1000 U/ml | 93 | 83 | 1 (reference) | 1 (reference) |  | 68 | 1 (reference) | 1 (reference) |
| ≥1000 U/ml | 107 | 100 | 1.56 (1.16-2.10) | 1.88 (1.36-2.61) |  | 87 | 1.55 (1.13-2.14) | 1.88 (1.31-2.70) |
|  |  |  |  |  |  |  |  |  |
| <500 U/ml | 77 | 70 | 1 (reference) | 1 (reference) |  | 58 | 1 (reference) | 1 (reference) |
| ≥500 U/ml | 123 | 113 | 1.35 (1.00-1.83) | 1.56 (1.11-2.19) |  | 97 | 1.30 (0.94-1.81) | 1.52 (1.05-2.21) |
|  |  |  |  |  |  |  |  |  |

* The multivariable, stage (stage III vs. stage IV)-stratified Cox regression model initially included age (continuous), sex (female vs. male), KPS (70-80 vs. 90-100), prediagnosis body mass index (continuous), tumor location (head/uncinate vs. body/tail vs. overlapping sites), diabetes mellitus (absent vs. present), chemotherapy regimen (Gemcitabine monotherapy vs. Gemcitabine plus 5-Fu vs. Gemcitabine plus nab-PTX vs. Gemcitabine plus DDP vs. Nab-PTX plus S-1), and radiotherapy (yes vs. no). A backward elimination with a threshold of *P* = 0.05 was used to select variables in the final models.

† CA19-9 change = ([CA19-9 at week-6]-[CA19-9 at baseline]) / (CA19-9 at baseline); per unit equals a 100% decrease.

Abbreviations: CI, confidence interval; HR, hazard ratio; KPS, Karnofsky Performance Status; PFS, progression-free survival; OS, overall survival.

Table S2. Survival by category of CA19-9 and platelet level in pancreatic cancer patients (CA19-9>37 U/ml)

|  |  |  | PFS | |  |  | OS | |
| --- | --- | --- | --- | --- | --- | --- | --- | --- |
|  | No. of cases | No. of events | Univariate  HR (95% CI) | Multivariate  HR^*^ (95% CI) |  | No. of events | Univariate  HR (95% CI) | Multivariate  HR^*^ (95% CI) |
| Baseline CA19-9 level |  |  |  |  |  |  |  |  |
| <8508 U/ml | 124 | 108 | 1 (reference) | 1 (reference) |  | 91 | 1 (reference) | 1 (reference) |
| >8508 U/ml | 52 | 51 | 1.80 (1.28-2.52) | 1.81 (1.24-2.64) |  | 45 | 1.99 (1.38-2.86) | 2.02 (1.35-3.02) |
|  |  |  |  |  |  |  |  |  |
| Change in CA19-9 level at week-6† |  |  |  |  |  |  |  |  |
| Per unit decreases of CA19-9 | 176 | 159 | 0.86 (0.78-0.94) | 0.85 (0.77-0.94) |  | 136 | 0.87 (0.79-0.97) | 0.84 (0.75-0.93) |
|  |  |  |  |  |  |  |  |  |
| Baseline Platelet level |  |  |  |  |  |  |  |  |
| Tertile 1 (lowest) | 58 | 51 | 1 (reference) | 1 (reference) |  | 45 | 1 (reference) | 1 (reference) |
| Tertile 2 | 58 | 55 | 1.19 (0.81-1.74) | 1.05 (0.70-1.57) |  | 45 | 0.92 (0.61-1.40) | 0.77 (0.50-1.20) |
| Tertile 3 (highest) | 60 | 53 | 0.95 (0.64-1.39) | 0.94 (0.63-1.42) |  | 46 | 0.82 (0.54-1.24) | 0.78 (0.51-1.21) |
|  |  |  |  |  |  |  |  |  |

* The multivariable, stage (stage III vs. stage IV)-stratified Cox regression model initially included age (continuous), sex (female vs. male), KPS (70-80 vs. 90-100), prediagnosis body mass index (continuous), tumor location (head/uncinate vs. body/tail vs. overlapping sites), diabetes mellitus (absent vs. present), chemotherapy regimen (Gemcitabine monotherapy vs. Gemcitabine plus 5-Fu vs. Gemcitabine plus nab-PTX vs. Gemcitabine plus DDP vs. Nab-PTX plus S-1), and radiotherapy (yes vs. no). A backward elimination with a threshold of *P* = 0.05 was used to select variables in the final models.

† CA19-9 change = ([CA19-9 at week-6]-[CA19-9 at baseline]) / (CA19-9 at baseline); per unit equals a 100% decrease.

Abbreviations: CI, confidence interval; HR, hazard ratio; KPS, Karnofsky Performance Status; PFS, progression-free survival; OS, overall survival.

Table S3 Changes in the CA19-9 and survival in relation to baseline platelet level in advanced pancreatic cancer patients

(CA19-9>37 U/ml)

|  |  |  | PFS per unit decrease of CA19-9† | |  |  | OS per unit decrease of CA19-9† | |
| --- | --- | --- | --- | --- | --- | --- | --- | --- |
|  | No. of cases | No. of events | Univariate  HR (95% CI) | Multivariate  HR^*^ (95% CI) |  | No. of events | Univariate  HR (95% CI) | Multivariate  HR^*^ (95% CI) |
| Baseline platelet level |  |  |  |  |  |  |  |  |
| Tertile 1 (lowest) | 58 | 51 | 0.71 (0.57-0.88) | 0.50 (0.37-0.68) |  | 45 | 0.59 (0.47-0.76) | 0.41 (0.29-0.58) |
| Tertile 2 | 58 | 55 | 0.60 (0.40-0.89) | 0.50 (0.26-0.98) |  | 45 | 0.71 (0.49-1.02) | 0.67 (0.42-1.06) |
| Tertile 3 (highest) | 60 | 53 | 0.90 (0.79-1.03) | 0.92 (0.79-1.07) |  | 46 | 0.93 (0.81-1.08) | 0.89 (0.74-1.06) |
|  |  |  |  |  |  |  |  |  |
| *P*_interaction_‡ |  |  | 0.015 | 0.012 |  |  | 0.009 | < 0.001 |
|  |  |  |  |  |  |  |  |  |

* The multivariable, stage (stage III vs. stage IV)-stratified Cox regression model initially included age (continuous), sex (female vs. male), KPS (70-80 vs. 90-100), prediagnosis body mass index (continuous), tumor location (head/uncinate vs. body/tail vs. overlapping sites), diabetes mellitus (absent vs. present), chemotherapy regimen (Gemcitabine monotherapy vs. Gemcitabine plus 5-Fu vs. Gemcitabine plus nab-PTX vs. Gemcitabine plus DDP vs. Nab-PTX plus S-1), and radiotherapy (yes vs. no). A backward elimination with a threshold of *P* = 0.05 was used to select variables in the final models.

† CA19-9 change = ([CA19-9 at week-6]-[CA19-9 at baseline]) / (CA19-9 at baseline); per unit equals a 100% decrease.

‡ *P*_interaction_ was calculated using the Wald test for the cross-product of prediagnosis platelet level (continuous) and CA19-9 decrease (continuous) in Cox regression model.

Abbreviations: CI, confidence interval; HR, hazard ratio; KPS, Karnofsky Performance Status.

Table S4 The interaction between changes in CA19-9 and baseline hematimetric variables in survival anlysis

|  |  |  | OS per unit decrease of CA19-9† | |
| --- | --- | --- | --- | --- |
|  | No. of cases | No. of events | Univariate | Multivariate |
| Neutrophils |  |  |  |  |
| *P*_interaction_‡ | 200 | 155 | 0.11 | 0.25 |
| Lymphocytes |  |  |  |  |
| *P*_interaction_‡ | 200 | 155 | 0.27 | 0.32 |
| NLR |  |  |  |  |
| *P*_interaction_‡ | 200 | 155 | 0.25 | 0.36 |
| PLR |  |  |  |  |
| *P*_interaction_‡ | 200 | 155 | 0.24 | 0.55 |

* The multivariable, stage (stage III vs. stage IV)-stratified Cox regression model initially included age (continuous), sex (female vs. male), KPS (70-80 vs. 90-100), prediagnosis body mass index (continuous), tumor location (head/uncinate vs. body/tail vs. overlapping sites), diabetes mellitus (absent vs. present), chemotherapy regimen (Gemcitabine monotherapy vs. Gemcitabine plus 5-Fu vs. Gemcitabine plus nab-PTX vs. Gemcitabine plus DDP vs. Nab-PTX plus S-1), and radiotherapy (yes vs. no). A backward elimination with a threshold of P = 0.05 was used to select variables in the final models.

† CA19-9 change = ([CA19-9 at week-6]-[CA19-9 at baseline]) / (CA19-9 at baseline); per unit equals a 100% decrease.

‡ Pinteraction was calculated using the Wald test for the cross-product of prediagnosis hematimetric variables level (continuous) and CA19-9 decrease (continuous) in Cox regression model.
